# Supplementary material for: Tailoring spin defects in diamond by lattice charging
Source: Nat Commun. 2017 May 17;8:15409. doi: 10.1038/ncomms15409 (PMC5442357; doi:10.1038/ncomms15409)
Supplement: Supplementary Information — Supplementary Figures, Supplementary Notes and Supplementary References. [file ncomms15409-s1.pdf]

Supplementary Information:
Tailoring spin defects in diamond by lattice charging

Felipe Fávaro de Oliveira,[1,*] Denis Antonov,[1] Ya Wang,[1]
Philipp Neumann,[1] Seyed Ali Momenzadeh,[1] Timo Häußermann,[1]
Alberto Pasquarelli,[2] Andrej Denisenko,[1,†] and Jörg Wrachtrup[1,3]

[1]*3. Institute of Physics, Research Center SCoPE and IQST, University of Stuttgart, 70569 Stuttgart, Germany*

[2]*Institute of Electron Devices and Circuits, University of Ulm, 89081 Ulm, Germany*

[3]*Max Planck Institute for Solid State Research, 70569 Stuttgart, Germany*

* f.favaro@physik.uni-stuttgart.de

† a.denisenko@physik.uni-stuttgart.de

Suplementary Note 1. NUMERICAL SIMULATIONS

Suplementary Note 1.1. Implantation process: Molecular Dynamics simulation

Similarly to Ref. [1], the Molecular Dynamics (MD) simulation is utilized to investigate events of nitrogen implantation in diamond. The ITAP MD (IMD) Program package[2, 3] is used in order to study the damage radius introduced by a single nitrogen atom initially accelerated to 4.0 keV (in z direction) in a $5.5 \times 5.5 \times 52\,\mathrm{nm}^3$ diamond structure. The atomic interaction is represented by a combination of a bond ordered Tersoff[4–8] and for decreasing atom separation (r < 0.3 Å) a two-body Ziegler-Biersack-Littmark (ZBL)[9, 10] potentials. In a first step, the diamond system is brought into equilibrium at room temperature. Next, the nitrogen atom is placed on top of the diamond surface ([100]-orientation) at a distance d= 2.51Å, which ensures its location beyond the action range of the Tersoff potential. The nitrogen atom is then implanted with a kinetic energy of $E_\mathrm{k} = 4.0\,\mathrm{keV}$ (normal to the surface). In order to provide enough statistics, individual implantation events with random starting positions on the xy-plane are repeated 120 times. In this way, the ion channeling effect on the stopping depth is taken into account, as can be observed by the resulting ion tracks presented in Supplementary Figure 1a and also discussed in the main text in figure 1a.

The vacancy distribution around the final position after implantation gives insight about the diameter of the damaged region (Δx in Supplementary Figure 1a) in the vicinity of the implanted atom. Supplementary Figure 1b shows the average size of such damaged region containing a high concentration of single vacancies (referred as "defect-clusters" in the main text) as a function of depth. As seen, this size is negligible at the ion channeling depth range: as explored in the main text, a negligible number of di-vacancies is formed in this region. At regions where ion channeling is negligible, however, a strong depth dependency is observed, leading to a higher number of di-vacancies around each NV center at individual defect-clusters. This correlation is further evidenced in Supplementary Figure 1c by nitrogen atomic profiles simulated by MD. The two off-angles 7° and 0° represent cases of suppressed and pronounced ion channeling, respectively. As illustrated in the correlation of Supplementary Figure 1 b and c, nitrogen atoms that rest closer to the diamond surface are surrounded by a higher concentration of single vacancies. This means that the formation of di-vacancies

occurs with higher probability within a depth range where ion channeling is suppressed.

Suplementary Note 1.2. Annealing process: kinetic Monte Carlo simulation

After implantation an additional annealing step is necessary to generate NV centers. In order to investigate the formation probability of NV centers and di-vacancies in their vicinity, kinetic Monte Carlo simulations are performed based on the simple hopping frequency of defects $\Gamma = \omega_{\mathrm{a}} e^{-\frac{E_{\mathrm{a}}}{k_{\mathrm{B}}T}}$ at a temperature of 950°C. Here, $\omega_{\mathrm{a}} = 10^{13}\mathrm{s}^{-1}$ denotes the frequency at which defects attempt to overcome a barrier of energy E_{a}. The values for activation energies are 2.3 eV for vacancy (V)[11, 12], 1.7 eV for interstitial nitrogen (N_I)[13] and 1.5 eV for carbon interstitial atoms (C_I)[14].

The Monte Carlo scheme models the diffusion process during annealing and investigates the corresponding influence on the conversion from implanted nitrogen atoms to NV centers (yield). The analysis includes the most important loss mechanisms of vacancies, such as migration to the surface, recombination with interstitials or the formation of di-vacancies and higher-order vacancy complexes. The process in which a self-interstitial atom replaces an on-site nitrogen at a lattice site and turns the latter into an interstitial nitrogen atom [12] was also included. In contrast to on-site nitrogen atoms which hardly move at 950°C, interstitial nitrogen atoms are mobile during annealing[12, 15]. The initial distribution after implantation are taken from MD simulations, as presented above. 100 annealing runs with different starting configurations were performed.

The simulation shows that the recombination of vacancies and interstitials happens within the first seconds of annealing since carbon interstitials are highly mobile. The number of vacancies that are left to possibly form NV centers is thus less than 50% of the number that is suggested by implantation simulations. The simulation of the vacancy diffusion within the crystal has completely come to an end after the simulated annealing time. On average, $> 90\%$ of the vacancies created by the implantation process are consumed after 2 hours of annealing (simulation time for the process). As seen in Supplementary Figure 2, approximately $30 - 40\%$ of implantation-induced single vacancies located within defect-clusters are expected to form di-vacancies, translating in $4 - 6$ di-vacancies surrounding NV centers in the close vicinity to the diamond surface (depth of < 5 nm). These values are used to discuss the experimental results in the main text.

Suplementary Note 1.3. NV-V_2 interaction Hamiltonian

To gain insight into the effect of di-vacancies, we use numerical calculations to obtain the resulting spin properties and magnetic noise characteristics of NV centers. In the calculation we place N_{V2} di-vacancies around a central NV center. The electronic spin of individual V_2 complexes (I = 1) are assumed to be coupled to each other and the central NV center electronic spin ($\mathbf{S}$) via dipole-dipole interaction. In the presence of a magnetic field of 33 mT aligned to the axis of the NV center (chosen as $\hat{z}$ axis), the Hamiltonian can be written as $H = H_{\rm NV} + H_{\rm int} + H_{\rm V_2}$, where the three terms correspond respectively to the central NV center, NV and V_2 hyperfine interactions and V_2 spins. These can be individually written as:

$$H_{\rm NV} = D_{\rm NV} S_{\rm z}^2 + \gamma_{\rm e} B S_{\rm z} \tag{1}$$

$$H_{\rm int} = S_{\rm z} \sum_j \mathbf{A}_{j,\rm z} . \mathbf{I}^j \tag{2}$$

$$H_{\rm V_2} = D_{\rm V_2} \sum_j \left(I_{\rm z}^j \right)^2 + \gamma_{\rm e} B \sum_j I_{\rm z}^j + \sum_{i,j} \mathbf{I}^i . \mathbf{D}_{ij} . \mathbf{I}^j \tag{3}$$

The zero-field splittings of the NV center and V_2 are largely different, being $D_{\rm NV} \approx 2.87\,\mathrm{GHz}$ and $D_{\rm V_2} \approx -0.31\,\mathrm{GHz}$, respectively[16]. The hyperfine tensor $\mathbf{A}_{j,z}$ and the dipole interaction tensor $\mathbf{D}_{i,j}$ depend on the spatial configuration of NV-V_2 spins. In a rotating frame defined by H_{NV}, the Hamiltonian can be rewritten as:

$$H = |0\rangle\langle 0| \otimes H_0 + |{+1}\rangle\langle{+1}| \otimes H_{+1} + |{-1}\rangle\langle{-1}| \otimes H_{-1} \tag{4}$$

where $H_0 = H_{\rm V_2}$ and $H_{\pm 1} = \pm \sum_j \boldsymbol{A}_{j,\rm z} \cdot \mathbf{I}^j + H_{\rm V_2}$. For the two levels $m_{\rm s} = 0$ and $m_{\rm s} = -1$ of interest here, the coherence decay is obtained through the following equations[17]:

$$T_{\rm cpmg_{2N+1}} = Tr(U_0^{'}(\tau) \underbrace{U_0^{'}(2\tau) U_{-1}^{'}(2\tau)}_{N} U_0^{'}(\tau) U_{-1}(\tau) \underbrace{U_0(2\tau) U_{-1}(2\tau)}_{N} U_0(\tau)), \tag{5}$$

$$T_{\rm cpmg_{2N+2}} = Tr(U_{-1}^{'}(\tau) \underbrace{U_0^{'}(2\tau) U_{-1}^{'}(2\tau)}_{N} U_0^{'}(2\tau) U_{-1}^{'}(\tau) U_0(\tau) U_{-1}(2\tau) \underbrace{U_0(2\tau) U_{-1}(2\tau)}_{N} U_0(\tau)) \tag{6}$$

The operators $U_0(\tau) = e^{-iH_0\tau}$ and $U_{-1}(\tau) = e^{-iH_{-1}\tau}$ describes the NV center spin state conditional to the evolution of V_2 spins. τ is the interval between the $\pi/2$ pulse and the first

π pulse. For the di-vacancy electronic spin bath with number $N_{V2} < 7$, the above equations can be calculated exactly. In order to simplify the calculation, only configurations where V_2 electronic spins are equidistantly distributed on a sphere of radius r_0 are considered (see Supplementary Figure 3a). For $N_{V2} = 3$ and 4, it is easy to see that the configuration are an equilateral triangle and a tetrahedron respectively. For $N_{V2} = 5$, there is indeed no obvious equidistant solution on a sphere. Here we use the configuration that two points sit at opposite poles and the other three points forming an equilateral triangle in the equatorial plane.

In addition, since a simple rotation of the sphere generates a new configuration due to the symmetry-breaker orientation of the NV center, an average over possible configurations finally gives the expected NV center T_2 time and CPMG-N decays, as exemplified in Supplementary Figure 3b for a number of four di-vacancies and $r_0 = 2\,\text{nm}$. As illustrated in Supplementary Figure 3c by the simulated values of T_2 times (Hahn-echo), the presence of a few di-vacancies in the nanometer vicinity of an NV center is enough to deplete the corresponding T_2 time to a few µs, as indeed seen in our and previous experiments. This indicates that di-vacancies are a dominant source of magnetic noise for NV centers created by low-energy nitrogen implantation, even in the nanometer vicinity of the diamond surface.

Figure 4 from the main text has presented results obtained by noise spectroscopy technique. As explained, NV centers created in the reference region suffer from an additional source of magnetic noise rather than surface spins. The experimental results were obtained by using decoherence decays from CPMG-32 measurements and noise spectral decomposition technique. The experimentally-extracted coupling strengths corresponding to the additional magnetic noise source were shown in figures 4b. Supplementary Figure 3d shows simulated values of coupling strengths arising from the presence of N_{V2} di-vacancies in the nanometer vicinity of a central NV center. We compare these values with experimental ones in the main text and conclude that the additional magnetic noise source in the reference region corresponds to the formation of di-vacancies complexes during thermal annealing.

Suplementary Note 1.4. Estimation of the effect of surface spins on near-surface NV centers

As discussed in the main text, the scaling of noise power spectral density with distance from the surface for NV centers in the sample area was attributed mainly to electron spins on the diamond surface. Such spins are related to localized electronic states at the diamond surface with a density of $\rho_{\mathrm{ss}} = 10^{13}\,\mathrm{cm}^{-2}$ (i.e. 1 spin per $10\,\mathrm{nm}^2$), as derived from our experimental results. Here we derive a simple model to address the experimental findings of the paper such as the amplitude and correlation time of observed magnetic noise. For simplicity we restrict to NV centers and surface spins with a quantization axis perpendicular to the surface. The modeled system is sketched in Supplementary Figure 4a.

Figure 4a in the main text shows the noise spectral density for an NV center located $r = 3.2\,\mathrm{nm}$ below the diamond surface in the sample area. Such an NV center interacts significantly with surface spins covering an area of about $10\,\mathrm{nm}^2$. The interaction decreases to zero for spins at the magic angle, which encircles an area of about $80\,\mathrm{nm}^2$. Thus, the average number of strongly coupled surface spins is roughly one.

For our estimation, we set the strongly coupled spin right above the NV center and we add a second spin with a distance d from it to mimic an alternating signal due to mutual flip-flops (see Supplementary Figure 4a). The flip-flop rate γ corresponds to the mutual magnetic dipole coupling and scales as $\gamma \approx 50\,\mathrm{MHz\,nm}^3/d^3$ for the considered arrangement. The correlation time of the corresponding noise is then $\tau_{\mathrm{c}} = \gamma^{-1}$. For the given spin density, we deduce the probability distribution to find a nearest neighbor spin at distance d as:

$$p(d) = 2\pi d \rho_{\mathrm{ss}} e^{-\pi \rho_{\mathrm{ss}} d^2}, \tag{7}$$

with $\int p(d)dd = 1$. As seen in Supplementary Figure 4b, the maximum probability occurs for a spin separation of $d \sim 1.3\,\mathrm{nm}$. Alternating fields by flip-flops are produced by anti-parallel aligned surface spin pairs and their total coupling Δ to the NV center, thus decreasing for smaller mutual distances d as:

$$\Delta(r,\theta) \approx \frac{50\,\mathrm{MHz\,nm}^3}{r^3}\left(3\cos^5\theta - \cos^3\theta - 2\right), \tag{8}$$

where the angle θ depends on d and r as $\tan\theta = d/r$, as sketched in Supplementary Figure 4a. The calculated coupling strength is shown in Supplementary Figure 4c as a function of

the separation of neighbor surface spins d.

From our current estimations we can derive an expected correlation between coupling strength Δ and correlation time τ_c, as presented in Supplementary Figure 4d by the blue line. In addition, the described probability distribution of surface spins is shown as a function of the expected correlation time (red line). Our model reproduces satisfactorily the experimentally obtained correlation times of $\sim 10\,\mathrm{ns}$ for NV centers in the sample area (see the blue dot in Supplementary Figure 4d), but underestimates the coupling strength. Potential reasons for this are our restrictions imposed by the orientation of the NV center, by including only two surface spins in our calculations and additional noise sources (i.e. P1 centers) as indicated in figure 4b of the main text.

Suplementary Note 2. LIMITS OF THE P$^+$-I JUNCTION STRUCTURE: CHARGE COMPENSATION BY IMPLANTATION-INDUCED DEFECTS

As described in the main text, the implantation of nitrogen with different energies in the space charge layer induced by the p$^+$-i junction structure has shown approximately a twofold yield enhancement. We investigate the behavior of the yield enhancement for different ion doses, implantation energies, as well as by using two diamonds with different thicknesses of the boron-doped layer ($d_{\mathrm{p+}} = 6\,\mathrm{nm}$, etched for 7 nm - diamond 1 and $d_{\mathrm{p+}} = 12\,\mathrm{nm}$, etched for 17 nm - diamond 2), as described in the main text. Supplementary Figure 5a shows the simulated profiles of holes (for both diamonds) together with atomic profiles of nitrogen atoms implanted with the three mentioned energies simulated by CTRIM on a [100]-oriented diamond surface (3°-off implantation angle). As depicted, the boron-doped layer of diamond 1 (thickness of 6 nm) is transparent to the nitrogen implantation in all three cases, whereas the diamond 2 (thickness of 12 nm) is transparent only to the 5.0 and 9.8 keV cases.

Supplementary Figure 5b shows the yield enhancement for the three energies and both diamonds as a function of ion fluence. Similar values ($\sim 100\%$) are observed for all cases. One interesting feature is the fact that this enhancement in yield decreases for increasing ion fluences, as evidenced by the results from diamond 1 shown in Supplementary Figure 5b. As discussed in the main text, we attribute this to the charge compensation in the substrate area due to implantation-induced defects. No compensation is observed for the diamond 2 for the cases of 5.0 and 9.8 keV (Supplementary Figure 5b with complementary data from diamond 2) since the concentration of implantation defects in the substrate within the used ion fluence range is not sufficient to compensate the holes.

This is further supported by the simulation results in Supplementary Figure 6 (as described in the "Methods" section - "Numerical simulations"), showing the charge compensation effect by additional donors across the p$^+$-i junction region. There, the profile of donors represent electronic states related to defects induced by nitrogen implantation at approximately the depth range as given by CTRIM simulations of nitrogen atomic profiles. For ion fluences below the critical value ($N_{\mathrm{D}(1)}$ for 5.0 and 9.8 keV cases in Supplementary Figure 6 b and d, respectively), the space charge layer induced in the substrate ($p_{(1)}$ for 5.0 and 9.8 keV cases in Supplementary Figure 6 b and d, respectively) remains unaffected. In this case, implantation-induced vacancies are efficiently charged during thermal annealing.

For ion fluences above the critical value ($N_{\mathrm{D}(2)}$ for 5.0 and 9.8 keV cases in Supplementary Figure 6 b and d, respectively), the space charge layer ($p_{(2)}$ for 5.0 and 9.8 keV cases in Supplementary Figure 6 b and d, respectively) shows a slight compensation for the case of 5.0 keV (reduction in concentration), whereas for the case of 9.8 keV a strong compensation is observed.

Suplementary Note 3. STATISTICS ON SPIN COHERENCE TIMES

The histogram in Supplementary Figure 7 presents the statistics on spin coherence times from NV centers in both sample and reference areas of two different fabricated diamonds. For the first diamond, the lower case in Supplementary Figure 7, T_2 times from NV centers created by nitrogen implantation with 5.0 keV of energy and an ion fluence of 10^{10} cm^{-2} are shown (same diamond used to produce the data shown in figure 3 in the main text). The thickness of the boron-doped layer in the sample area was initially 6 nm. These measurements were performed on the bare oxygen-terminated surface after the final plasma etching step of 7 nm (etching performed on both regions). Although the depth of the majority of these NV centers is unknown, in average, the sample area shows much longer T_2 times in comparison to the reference area. A careful analysis of the histogram reveals that NV centers in the sample area show T_2 times that can be classified into two groups: the first group with values < 60 µs and the second group with values $85\,\mu\text{s} < T_2 < 160\,\mu\text{s}$. We attribute the first and second groups to NV centers formed within the ion stopping (shorter T_2 times, closer to the diamond surface) and ion channeling (longer T_2 times, deeper) ranges, respectively.

It should be highlighted that this behavior is observed in several diamonds fabricated under similar conditions, with small deviations due to variations in crystal quality. As an example, the upper case of the histogram in Supplementary Figure 7 presents T_2 times from NV centers created in a second diamond by molecular nitrogen ($^{14}N_2^+$) implantation with 30 keV of energy and an ion fluence of 4×10^9 atoms cm^{-2}. The thickness of the boron-doped layer in the sample area was initially 30 nm. Similarly as in the other fabricated diamond (see the discussion of figure 3c in the main text), this thickness was set to match the tail of the ion stopping range according to CTRIM simulations. For this implantation event, an incident angle of 7° was chosen in order to reduce the probability of ion channeling. These measurements were performed on the bare oxygen-terminated surface after the final plasma etching step of 34 nm (etching performed on both regions). As seen in the histogram, T_2 times from NV centers in the sample area in both fabricated diamonds are much longer in comparison to those in the reference area. This assures the reproducibility of the technique and the observed enhancements. The presented results also further highlight the manipulation of the charge state of implantation defects as a universal tool, even if experimental conditions must be changed to fulfill the needs of different NV-based applications, i.e. higher

implantation energies for the creation of deeper NV centers in the crystal. Moreover, the T_2 times from NV centers in the reference areas of both fabricated diamonds were comparably low. This indicates that residual implantation defects are indeed the dominant source of spin decoherence in the case of standard nitrogen implantation followed by thermal annealing.

As described in the "Methods" section of the main text, the depth of each individual NV center for the case of the diamond implanted with 5.0 keV of energy was measured by means of T_1 relaxation induced by Gd^{3+} ions located on the diamond surface. From all the NV centers initially measured for the lower presented data in Supplementary Figure 7, only those that fulfill the criteria of (i.) having a spin contrast (i.e. Rabi oscillations) above 15% and (ii.) showing stable photon emission during all three rounds of measurements for the depth calibration (bare surface - layer of Gd^{3+} ions - cleaned surface) were taken into account. The majority of the NV centers in the reference area (approximately 70%) and a parcel of the NV centers in the sample area (approximately 20%) demonstrated low spin contrast and/or photo-blinking. These two features result in a low signal-to-noise ratio, leading to low precision and longer integration times in the needed spin measurements, specially in the T_1 times (if measurable at all). These NV centers were therefore excluded from the data presented in the main text (figure 3). We attribute this blinking behavior and/or low spin contrast to the presence of electronic states in the band gap induced by lattice defects that would change the charge state of nearby NV centers. These defects might be generated not only by the implantation and annealing processes, but also by the surface polishing[18] and during the crystal growth. In particular, charge exchange between Gd^{3+} ions and the diamond surface would also result into photo-instability and low spin contrasts due to higher NV^0/NV^- rates. The T_2 and T_1 times of NV centers that fulfill the above mentioned criteria are shown in figure 3 a) and b) in the main text, respectively.

It must be emphasized that, although the data points from the reference area at depths of ~ 3 nm in figure 3 in the main text show extremely short T_2 times, T_1 times of a few milliseconds were measured. This behavior is a typical signature from the presence of electron spins in the vicinity of NV centers[19, 20]. Electron spin flip-flops produce a typical magnetic noise spectral density with maximum values in the kHz-MHz frequency range (where the T_2 times measured by Hahn-echo scheme are sensitive), but with negligible values at the GHz range (where the T_1 times are sensitive). This reinforces the attribution that di-vacancy electron spins are a dominant source of spin decoherence, as discussed in the main text.

SUPPLEMENTARY REFERENCES

[1] Antonov, D. *et al.* Statistical investigations on nitrogen-vacancy center creation. *Applied Physics Letters* **104**, 012105 (2014).

[2] Roth, J., Gähler, F. & Trebin, H.-R. A molecular dynamics run with 5180116000 particles. *Int. J. Mod. Phys. C: Computational Physics & Physical Computation* **11**, 317 (2000).

[3] Stadler, J., Mikulla, R. & Trebin, H.-R. Imd: A software package for molecular dynamics studies on parallel computers. *Int. J. Mod. Phys. C* **8**, 1131–1140 (1997).

[4] Tersoff, J. New empirical approach for the structure and energy of covalent systems. *Phys. Rev. B* **37**, 6991–7000 (1988).

[5] Tersoff, J. Empirical interatomic potential for carbon, with applications to amorphous carbon. *Phys. Rev. Lett.* **61**, 2879–2882 (1988).

[6] Tersoff, J. Empirical interatomic potential for silicon with improved elastic properties. *Phys. Rev. B* **38**, 9902–9905 (1988).

[7] Tersoff, J. New empirical model for the structural properties of silicon. *Phys. Rev. Lett.* **56**, 632–635 (1986).

[8] Tersoff, J. Modeling solid-state chemistry: Interatomic potentials for multicomponent systems. *Phys. Rev. B* **39**, 5566–5568 (1989).

[9] Ziegler, J., Biersack, J. & Littmark, U. *SRIM, the Stopping and Range of Ions in Matter*, vol. 1 (Pergamon Press, 1985).

[10] Chan, H. *et al.* The effect of interatomic potential in molecular dynamics simulation of low energy ion implantation. *Nucl. Instr. Meth. Phys. Res. Sec. B: Beam Interactions with Materials and Atoms* **228**, 240 – 244 (2005).

[11] Davies, G., Lawson, S. C., Collins, A. T., Mainwood, A. & Sharp, S. J. Vacancy-related centers in diamond. *Phys. Rev. B* **46**, 13157–13170 (1992).

[12] Mainwood, A. Nitrogen and nitrogen-vacancy complexes and their formation in diamond. *Phys. Rev. B* **49**, 7934–7940 (1994).

[13] Goss, J. P., Briddon, P. R., Papagiannidis, S. & Jones, R. Interstitial nitrogen and its complexes in diamond. *Phys. Rev. B* **70**, 235208 (2004).

[14] Breuer, S. J. & Briddon, P. R. *Ab initio* investigation of the native defects in diamond and self-diffusion. *Phys. Rev. B* **51**, 6984–6994 (1995).

[15] Field, J. *The Properties of natural and synthetic diamond* (Academic Press, 1992).

[16] Twitchen, D. J., Newton, M. E., Baker, J. M., Anthony, T. R. & Banholzer, W. F. Electron-paramagnetic-resonance measurements on the divacancy defect center $r4/w6$ in diamond. *Phys. Rev. B* **59**, 12900–12910 (1999).

[17] Yang, W. & Liu, R.-B. Quantum many-body theory of qubit decoherence in a finite-size spin bath. *Phys. Rev. B* **78**, 085315 (2008).

[18] Volpe, P.-N. *et al.* Defect analysis and excitons diffusion in undoped homoepitaxial diamond films after polishing and oxygen plasma etching. *Diamond and Related Materials* **18**, 1205 – 1210 (2009).

[19] Bar-Gill, N. *et al.* Suppression of spin-bath dynamics for improved coherence of multi-spin-qubit systems. *Nat. Commun.* **3**, 858 (2012).

[20] Myers, B. A. *et al.* Probing surface noise with depth-calibrated spins in diamond. *Phys. Rev. Lett.* **113**, 027602 (2014).

FIGURES

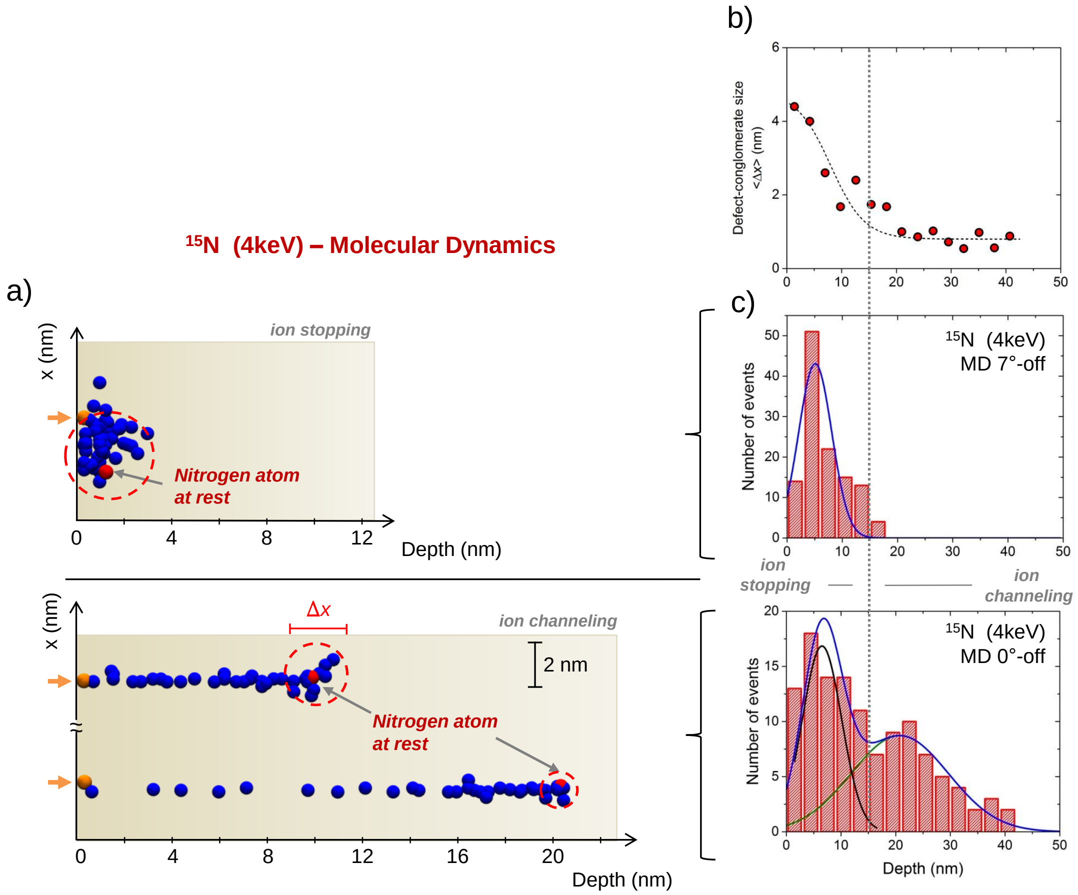

Supplementary Figure 1. **Implantation-induced defect-clusters around individual NV centers by numerical simulations. a)** Three examples of ion tracks resulting from simulated impacts of individual nitrogen atoms in the diamond lattice with the same energy (4.0 keV, Molecular Dynamics simulations), but different contributions of ion channeling. The size (Δx) of the volume containing the cluster of single vacancies that surrounds each implanted nitrogen atom is depicted by the red-dashed circles. **b)** The size of such defect-clusters is shown as a function of nitrogen penetration depth. **c)** Nitrogen atomic profiles simulated by MD for a [100]-oriented diamond lattice (0°- and 7°-off angles represent dominating and fully suppressed ion channeling of implanted atoms, respectively). Solid lines are Gaussian fit functions.

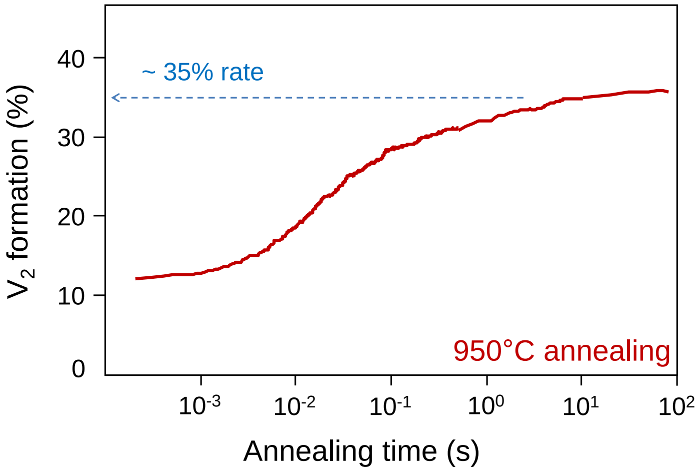

Supplementary Figure 2. **Kinetic Monte Carlo simulation of the formation of di-vacancies.** The formation probability from single vacancies to di-vacancies is plotted as a function of annealing time. As sketched, a saturation occurs at a value of approximately $30-40\%$ for annealing times $> 100\,\mathrm{s}$.

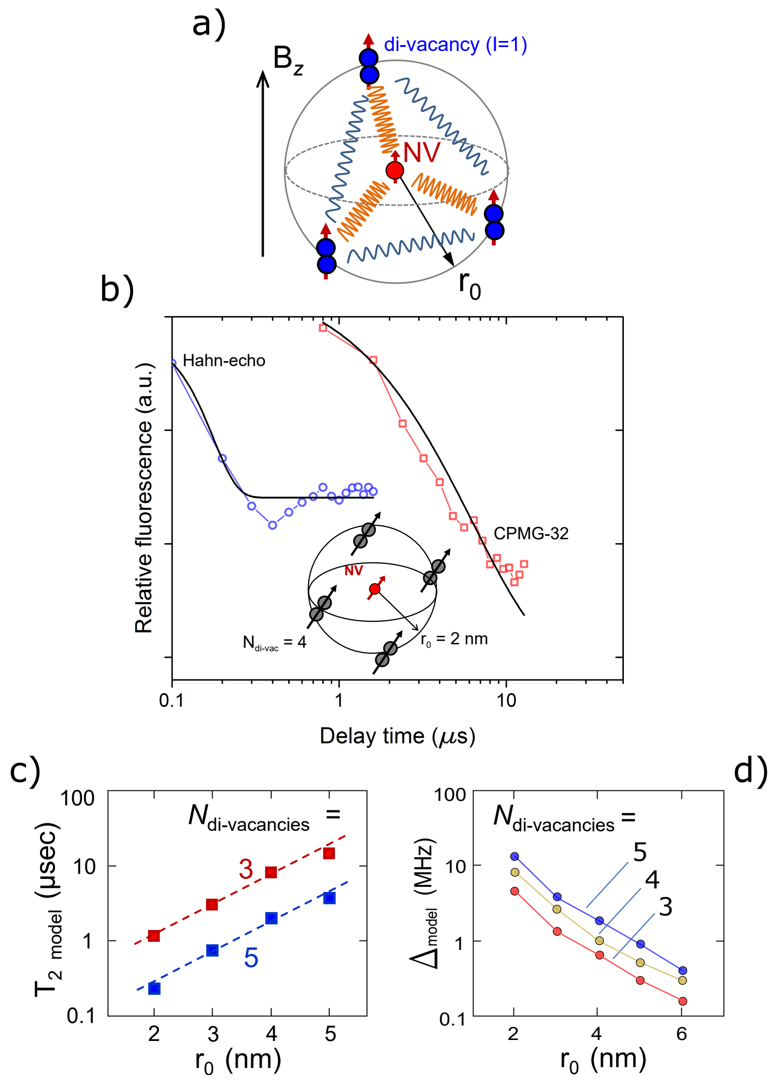

Supplementary Figure 3. **Numerical calculation on the NV-V_2 interaction. a)** Sketch of the 3D model used to simulate the presence of N di-vacancy electron spins equally distributed on a sphere with radius r_0 centered on a single NV center. **b)** Example of simulated decoherence decays (T_2 Hahn-echo and CPMG-32) for the case of $N_{V2} = 4$ di-vacancies and $r_0 = 2\,\text{nm}$. **c)** Calculated values of spin coherence times (Hahn-echo) for different number of di-vacancies as a function of r_0. **d)** Calculated values of the coupling strength related to the magnetic noise generated by N_{V2} di-vacancies at different r_0. These values correspond to the noise spectra calculated for the CPMG-32 decoherence decays.

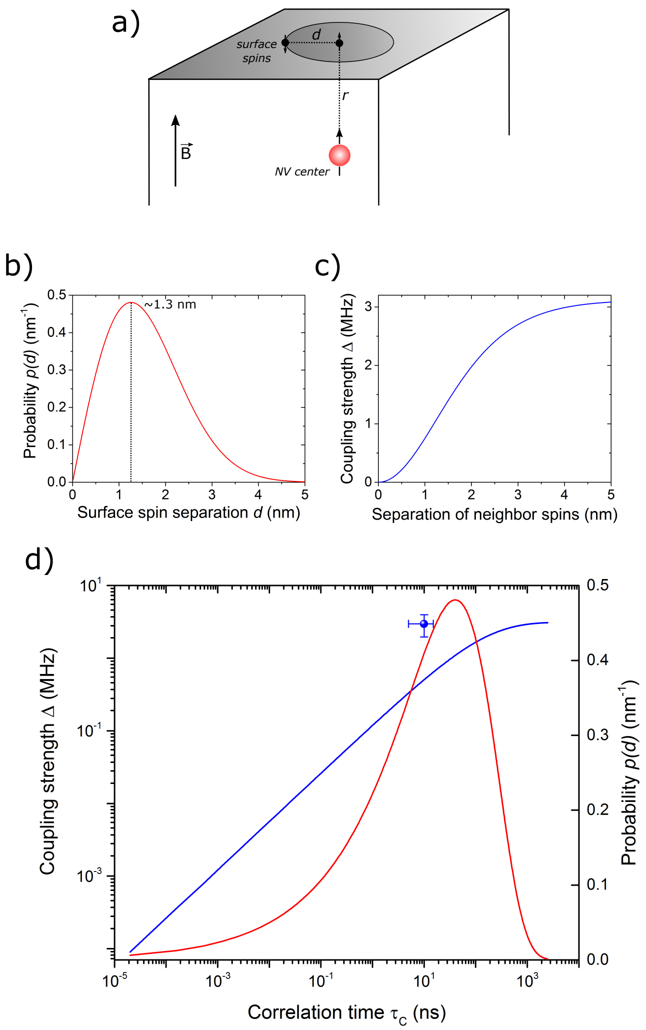

Supplementary Figure 4. **Numerical calculations of the surface spin bath. a)** Sketch of the modeled system consisting of an NV center located at a depth r with the main axis perpendicular to the surface (aligned to the external magnetic field $\mathbf{B}$) and surface spins with a density ρ_{ss} and mean separation d. **b)** Probability distribution versus the spin separation. The maximum probability of near-neighbor spins related to the calculated $\rho_{\mathrm{ss}} \sim 10^{13}$ spins cm^{-2} occurs for a separation of approximately 1.3 nm. **c)** Calculated NV-surface spins dipolar coupling strength versus the separation d of surface spins. **d)** NV-surface spins dipolar coupling strength (blue line, left axis) and probability distribution of neighbor surface spins (red line, right axis) versus the corresponding correlation time. The blue dot represents the experimentally extracted value of coupling strength and correlation time for an NV center with 3.2 nm of depth in the sample area, as shown in figure 4a in the main text. The error bars correspond to the uncertainty in the data fit.

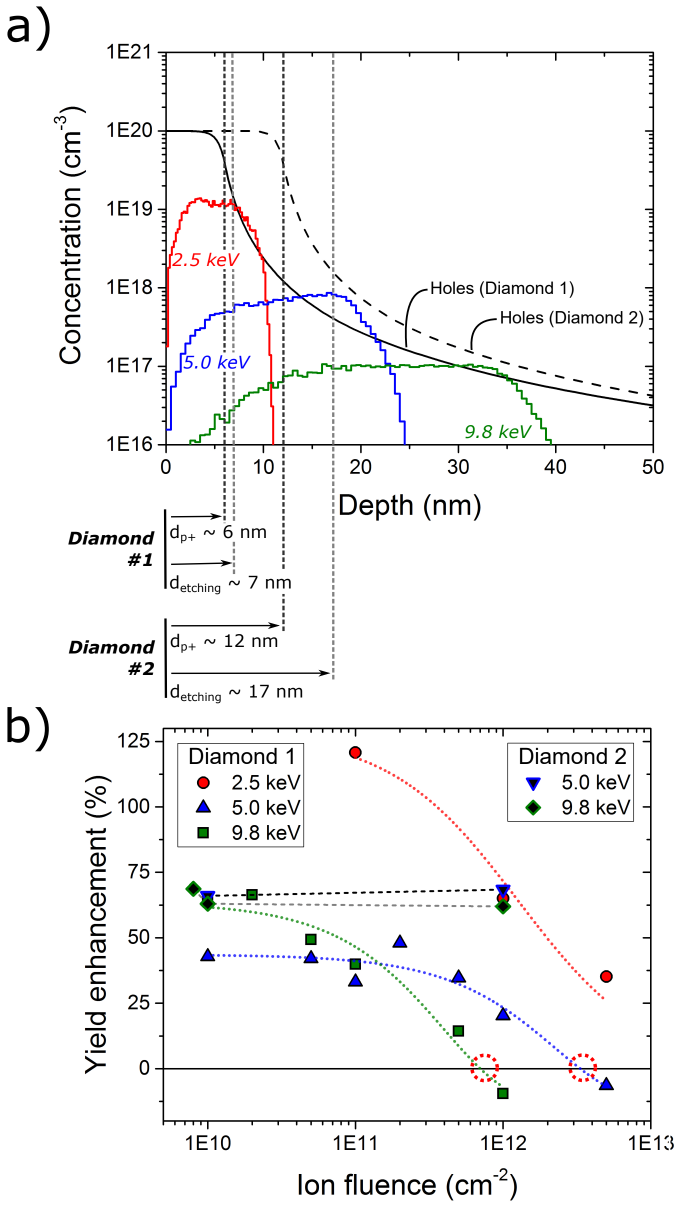

Supplementary Figure 5. **Charge compensation effect. a)** 1D depth profiles of holes (black-solid line for a $d_{p+} = 6\,\text{nm}$ and black-dashed line for a $d_{p+} = 12\,\text{nm}$). Atomic profiles for nitrogen atoms simulated by CTRIM for three different energies are shown for the critical ion fluences corresponding to a $d_{p+} = 6\,\text{nm}$ (see text) - $3 \times 10^{13}\,\text{cm}^{-3}$ for 2.5 keV, $3 \times 10^{12}\,\text{cm}^{-3}$ for 5.0 keV and $8 \times 10^{11}\,\text{cm}^{-3}$ for 9.8 keV. **b)** Enhancement in the yield of NV centers versus the ion fluence for the three mentioned implantation energies and two diamonds. The zero represents equal yields from the sample and reference regions.

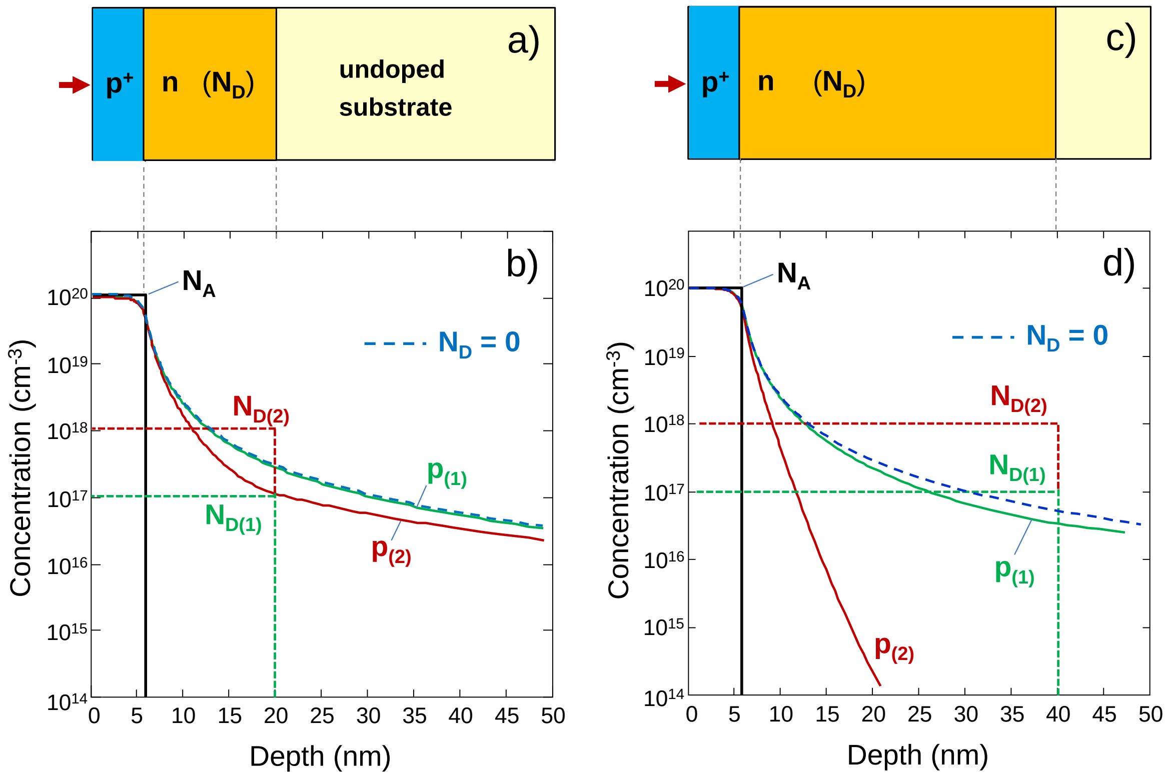

Supplementary Figure 6. **Charge compensation for the cases of 5.0 and 9.8 keV. a) and c)** Schematic cross sections of the junction structure formed at the critical ion fluence for the cases of 5.0 and 9.8 keV, respectively; the implantation-induced defects and P1 centers create an area acting as the host of donors/charge traps (p^+-n junction type). **b)** Corresponding space charge layers simulated for the cases of 5.0 keV with 3×10^{11} ($p_{(1)}$ - $N_{D(1)}$) and 3×10^{12} cm^{-2} ($p_{(2)}$ - $N_{D(2)}$) ion fluences. **d)** Corresponding space charge layers presented for the cases of 9.8 keV with 8×10^{10} ($p_{(1)}$ - $N_{D(1)}$) and 8×10^{11} cm^{-2} ($p_{(2)}$ - $N_{D(2)}$) ion fluences.

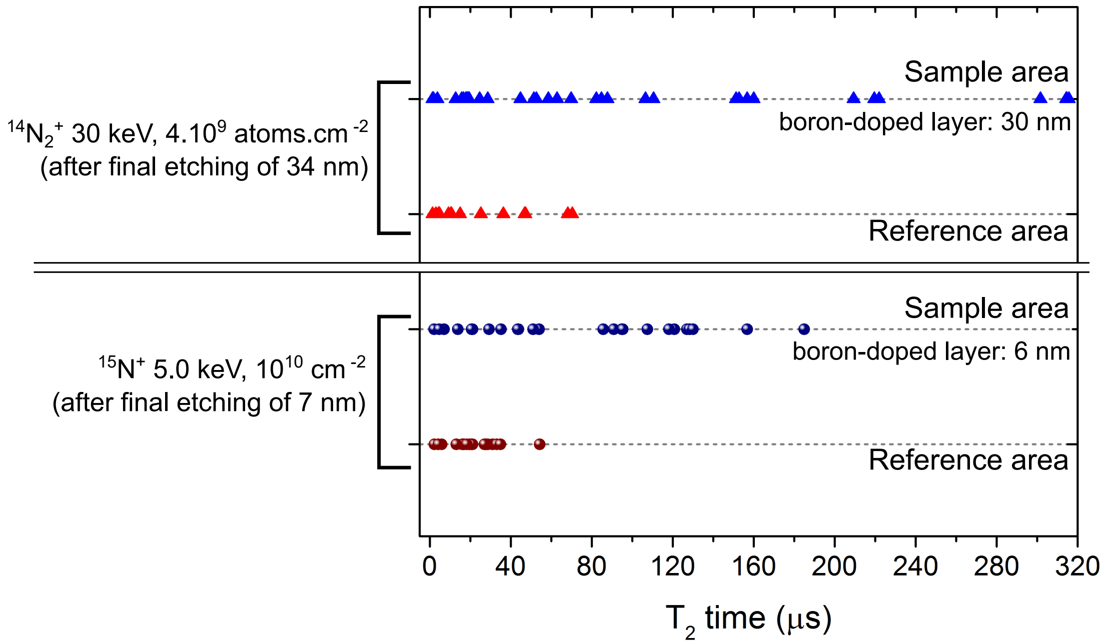

Supplementary Figure 7. **Statistics on the spin coherence times of NV centers located at the sample and reference areas of two different fabricated diamonds.** In the lower part, spin coherence times measured for ∼ 30 NV centers at the sample (red dots) and reference (blue dots) areas after the final etching step of 7 nm (5.0 keV of energy with an ion fluence of 10^{10} cm^{-2}, see the discussion of figure 3 in the main text for further details). In the upper part, spin coherence times measured for ∼ 35 NV centers at the sample (red triangles) and reference (blue triangles) areas for the case of molecular nitrogen (N_2^+) implantation with an incident angle of 7° and 30 keV of energy (ion fluence of 4×10^9 atoms cm^{-2}) after the final etching step of 34 nm. The boron-doped layer thickness in this case was initially 30 nm in the sample area. These measurements were performed with a magnetic field of approximately 33 mT aligned to the NV axis.
